# Seroepidemiology of respiratory syncytial virus infection in rural and semi-rural areas of the Littoral region of Cameroon

**DOI:** 10.1186/s12879-021-05838-w

**Published:** 2021-02-04

**Authors:** Henshaw Mandi, Bekolo Cavin Epie, Agnes Eyoh, Sindhiya Jan, Sue Ann Costa Clemens, Ralf Clemens, Solomon Yimer

**Affiliations:** 1 Coalition for Epidemic Preparedness Innovations CEPI, Oslo, Norway; 2 Institute for Public Health, University of Siena, Siena, Italy; 3 Department of Public Health, University of Dschang, Dschang, Cameroon; 4 National Early Infant Reference Laboratory, Mutengene, Cameroon; 5 Vismederi srl, Siena, Italy

**Keywords:** Respiratory syncytial virus, Epidemiology, Cameroon

## Abstract

**Background:**

The respiratory syncytial virus (RSV) has been established as a leading cause of acute lower respiratory illness (ALRI) in infants and children. In 2015, the global disease burden (GBD) study estimated that the overall RSV-ALRI mortality could be as high as 118,200, with most death occurring in low- and middle-incomes countries (LMIC). This study aimed to assess the burden of RSV infection among children less than 2 years with acute respiratory infections (ARI) in the Littoral region of Cameroon.

**Methods:**

We carried out a cross-sectional study in seven health centres in the Littoral region of Cameroon**.** Venous blood was collected using serum separation tubes from eligible children who visited these health centres with acute respiratory infections. ELISA (Enzyme-linked immunosorbent assay) testing was used to assess the seroprevalence of anti-IgM RSV for the total population and by selected demographic and health parameters and potential risk factors.

**Results:**

The overall RSV-associated ARI seroprevalence was 33% (95%CI:23.6–42.3; 33/100 children). The only demographic factor significantly associated with RSV acquisition was age of 6 months and below (odds ratio: 7.54 (2.62, 23.36); *p* = 0.000). Children who were clinically diagnosed to be concomitantly infected with malaria had a lower risk of RSV infection (odds ratio: 0.38 (0.14, 0.95; *P* = 0.03).

**Conclusions:**

The RSV burden is high among children less than 2 years with ARI in the Littoral region of Cameroon. There is a need for an effective public health RSV surveillance system with standard laboratory techniques and equipment to better understand the RSV disease age-specific incidence, seasonality, risk factors and RSV burden among patients in communities in Cameroon.

## Introduction

The respiratory syncytial virus (RSV) has been established as a leading cause of acute lower respiratory illness (ALRI) in infants and children [1, 2]. In 2015, the global disease burden (GBD) study estimated that the overall RSV-ALRI mortality could be as high as 118,200 (uncertainty range [UR] 94,000–149,400), with most death occurring in low- and middle-incomes countries (LMICs) [3]. In the same GBD study it was also estimated that 33∙1 million episodes of ALRI and 3∙2 million hospital admissions and 59,600 (48000–74,500) in-hospital deaths in children younger than 5 years were attributable to RSV worldwide [3]. Hall et al. in 2009 found a significant RSV disease burden in neonates, with estimates of 40 episodes per 1000 neonates per year (95% CI 2.5–635.7) [4]. Resource-limited countries have more than twice the incidence of severe disease seen in developed countries [5–7]. The need for precise epidemiological data of RSV as novel RSV therapeutics such as affordable monoclonal antibodies are reaching the final stages of development whilst RSV vaccines for use in a pediatric population are still early stages [8–10].

By 2 years of age, almost all children will have been infected with RSV, and approximately 50% infected twice. Re-infection with RSV can occur throughout life and is often symptomatic. Despite an increasing number of epidemiological studies of RSV-associated lower respiratory tract infections (LRTI) published in developing countries, there is still a need to establish a public health RSV surveillance system to improve the incidence estimates and to determine national and regional variations in RSV disease burden in countries [11]. Among children aged less than 5 years old, the incidence of RSV-associated LRTI per 1000 child-years was 34 in Indonesia, and 94 in Nigeria and the incidence of severe RSV-associated LRTI per 1000 child-years was 5 in Mozambique, 10 in Indonesia, and 9 in South Africa [12]. However, these surveillance systems are not uniform and hence data are difficult to compare.

A systematic review identified 20 studies that investigated 18 potential risk factors for RSV–associated ALRI in children younger than 5 years old. Eight risk factors were significantly associated with RSV–associated ALRI, namely preterm birth, low birth weight, male sex, having siblings, maternal smoking, history of atopy, no breastfeeding, and crowding [13].

In children, less than 5 years in Cameroon, low respiratory infections, malaria, diarrheal diseases, and nutritional deficiencies are the leading causes of morbidity and mortality. In children aged 2 months to 5 years, malaria (21%), diarrhea (17%), pneumonia (17%), and HIV/AIDS (7%) predominate mortality. In Cameroon in 2011 there were 189 health districts, 4034 health facilities of with 72% were public and 28% private health care providers. These health facilities serve the general population. Most of the services are still out-of-pocket and considered expensive by the public. The high cost of healthcare services in private health facilities encourage users to resort to informal care or home care [14]. Essential family practices and interventions with a high impact on the child’s health (vaccination, exclusive breastfeeding, etc.) are thus not, however, sufficiently implemented to reverse the figures, as mentioned above.

An earlier study in Cameroon showed that RSV circulates from the beginning of the dry season October to December and 5.7% of outpatients with influenza-like illness visiting the influenza surveillance centers in 2009 were diagnosed as being RSV infected [15]. Another study showed that RSV was the second most common respiratory virus (13.3%) after human adenovirus in children hospitalized in Yaoundé, Cameroon [16]. The unicentric study did not find a significant age-specific RSV prevalence, which might have underestimated the overall detection rate for selected viruses. The enrolled cases may not be representative of the entire population of children in Cameroon as these studies were based in urban settings. More studies are needed especially also expanding to rural or semi-urban areas to provide a better understanding of the epidemiology and spectrum of illness caused by respiratory viruses, especially RSV in Cameroon [16]. Therefore, we sought to assess the burden of RSV infection among children less than 2 years in rural and semi-rural areas of the Littoral region of Cameroon.

## Methods

### Study area

Cameroon, a country in Central Africa, has an area of 475,650 km^2^. According to the 3rd National Population and Housing Census, the estimated population of Cameroon in 2015 was at about 22,179,707 inhabitants. The annual crude birth rate in Cameroon is 35.4/1000 inhabitants, translating into about 882,000 birth annually [17]. Accessibility to public health facilities is more evident for the wealthiest segments of the people, like those living in urban areas (52%). The health sector is structured into primary, secondary, and tertiary levels [14].

**Fig. 1:**
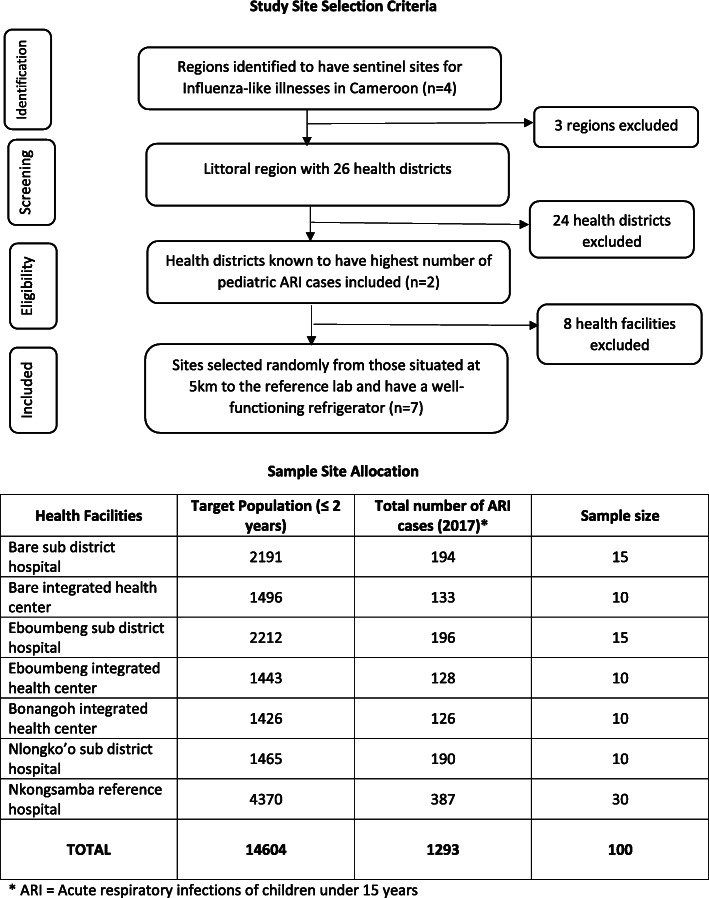
Study site selection and sample size allocation

The Littoral region of Cameroon which comprises of 26 health districts and 212 health facilities with an estimated total population of over 3 million inhabitants and almost 80,000 birth cohort. This study was conducted in seven health centres: the district hospital of Nkongsamba, Bare sub-district health centre, Eboumbeng sub-district health centre, Eboumbeng integrated health centre, Bonangoh integrated health centre, Nlongko’o sub-district health centre, and Bare integrated health centre. Regional Hospital of Nkongsamba represents a secondary hospital of about 320,000 inhabitants and also served as the reference hospital for all the other study sites in the rural communities. The rest of the study sites are part of the primary healthcare level. The site selection procedure for this study was as follows: firstly, we randomly the selected Littoral region among four areas that comprise sentinel sites for influenza-like illnesses based on regional health retrospective data. Secondly, we chose two health districts (Melong and Nkongsamba) among 26 health districts of the region. Thirdly, we selected at random the seven study sites from the list of 45 health centres in the two health districts respecting the selection criteria of being situated at most 5 km from the reference laboratory and possessing a well-functioning refrigerator (Fig. 1). The study sites are representative of the healthcare system in Cameroon.

### Sample size

In order to calculate the total number of patients required for estimating the prevalence of RSV precisely, we use normal approximation to binomial distribution. Specifically, first we assume that the true prevalence of RSV in outpatient and admitted patients is 50%, and there is a possibility of 3% non-response. Subsequently, in order to obtain a 95% confidence interval for RSV prevalence with a 10% margin of error, we will require 100 patients. The choice of 50% as the true prevalence was motivated by the fact that a prevalence of 50% requires the most number of patients. That is, it is a conservative choice. Consequently, if the true prevalence is either smaller or larger than 50%, then our margin of error will only be smaller than 10%, but never more than it.

The stratified sampling proportional allocation strategy was used to allocate the sample size to each study site. Each study site was considered a stratum and the size was based on the number of pediatric ARI cases of the year 2017 (Fig. 1).

### Study design, target population, and sampling

This multicentric cross-sectional study was conducted within 6 months, from March to September 2018. The primary objective of the study was to determine the prevalence of RSV infection in children less than 2 years of age with symptoms of ARI as measured by RSV ELISA IgM. Secondary objective was to assess the risk associated with various sociodemographic, medical and environmental characteristics and the probability of testing positive for RSV IgM. These characteristics and their categories are defined in Table 1.

**Table 1:** RSV IgM seropositivity and odds ratios for factors potentially associated with RSV IgM positivity

Variable	Groups	#RSV+	#RSV-	#Total (N)	O.R. (95% CI)	***p***-value
Health facility	Primary Health Center	12	23	100	1.09 (0.41, 2.82)	1
	Hospital	21	44			
Sex	Female	19	29	89	1.78 (0.67, 4.91)	0.26
	Male	11	30			
Birth weight	More than 2.5 kg	25	53	87	0.94 (0.18, 6.31)	1
	Less than 2.5 kg	3	6			
Age	≤ 6 months	18	9	100	7.54 (2.62, 23.36)	0
	> 6 months	15	58			
Chronic lung disease	Yes	1	7	99	0.27 (0.01, 2.22)	0.26
	No	32	59			
Previous wheezing	Yes	5	4	99	2.74 (0.54, 14.9)	0.16
	No	28	62			
Malaria	Yes	13	42	99	0.38 (0.14, 0.95)	0.03
	No	20	24			
Prematurity	Yes	5	14	98	0.73 (0.19, 2.45)	0.78
	No	26	53			
Maternal Education	Up to primary school	10	14	96	1.61 (0.55, 4.64)	0.33
	More than primary school	22	50			
Tobacco smoking in family	Yes	4	15	98	0.49 (0.11, 1.74)	0.28
	No	28	51			
Indoor air pollution	Yes	22	33	98	2.18 (0.84, 6.01)	0.09
	No	10	33			
Type of feeding	Only breastfeeding	9	4	84	5.6 (1.37, 27.82)	0,01
	Mixed	20	51			

*RSV* Respiratory syncytial virus; *O.R.* Odds ratio.

*The total (N) of patients is lower than 100 in some categories due to missing data. Odds: odds of having a being RSV IgM positive.*

A case of ARI was defined as illness fulfilling age-specific clinical inclusion criteria with onset within 7 days in a child aged less than 2 years. Acute respiratory infection was defined as an illness presenting with one or more of the following symptoms: fever, cough, earache, nasal congestion, rhinorrhea, sore throat, vomiting after coughing, wheezing, and labored, rapid, or shallow breathing. Children who had respiratory symptoms lasting more than 14 days because RSV infection may have been acquired in the health facility during the perinatal period, who had neutropenia from chemotherapy, had been hospitalized elsewhere within 4 days or were newborns who had been hospitalized since birth were not enrolled.

The study was approved by a national ethical review committee. Regulatory approval was not sought as this was not an interventional drug or vaccine study. Mothers or caretakers of the children were invited to participated in the study, and after obtaining written informed consent children were enrolled. Study relevant data such as clinical signs and symptoms, sociodemographic, medical and environmental information were entered by study nurses into a questionnaire (Table 1). Nurses of each study health facility were previously trained in data collection and documentation using a pilot-tested questionnaire.

Venous blood of 2-ml was collected from all enrolled patients centrifuged and plasma transferred into 1-ml cryotubes containing virus transport medium. Plasma was stored at 4–8 °C at the collection site for a maximum of 48 h. Laboratory technicians were trained prior to study start in blood sample collection, transportation, and storage procedure as per a written Laboratory manual. Samples were then transported, maintaining the cold chain using the triple packaging system to the testing site and stored at − 80 °C pending testing. The Abcam ELISA (ab108766 anti-RSV IgM Human, Abcam, Cambridge, United Kingdom) testing procedure: A 96-well plate was precoated with Respiratory syncytial virus antigens to bind cognate antibodies. One hundred μL of controls or diluted sample were added into appropriate wells and incubated at 37 °C. Following washing, a horseradish peroxidase (HRP) labeled anti-Human IgM conjugate was added to the wells, which binds to the immobilized Respiratory syncytial virus-specific antibodies. 3,3′,5,5′-Tetramethylbenzidine (TMB) was then catalyzed by the HRP to produce a blue substrate that changes to yellow after adding an acidic stop solution. The yellow coloration is directly proportional to the amount of Respiratory syncytial virus IgM sample captured in plate.

### Data collection and analysis

Data documented in the questionnaires and laboratory results were recorded and stored in a spread sheet using MS Excel. The statistical analysis was performed using Stata, version 11.0 (StataCorp, College Station, TX).

The primary outcome - RSV prevalence - was determined by proportion of RSV IgM positive children, calculated by dividing the total number of RSV IgM positive children by the total number of children tested for RSV IgM. The corresponding 95% confidence interval was obtained using normal approximation to binomial distribution.

A second analysis assessed the association between the clinical characteristics and their categories as defined in Table 1 and the probability of testing positive for RSV IgM. Each clinical characteristic is categorical. Hence, to analyze them we utilized fisher’s exact test. The null hypothesis in each test was that the odds of obtaining a positive RSV test in each of the two groups of a clinical characteristic were equal. The alternative hypothesis was that the odds were unequal. A *p*-value of < 0.05 was considered statistically significant.

## Results

The overall RSV-associated ARI seroprevalence was 33% (95%CI:23.6–42.3; 33/100 children). Clinically, 10/33 of these RSV-positive children presented with a severe bronchiolitis and 5/33 with pneumonia as diagnosed by the attending physicians.

In total, 100 eligible children were enrolled, with (65) 65% of cases coming from hospitals. The male-to-female ratio was 0.85:1, with a mean age of 10.6 months [Standard deviation (SD) = 6.11]. Twenty-seven of the study subjects were infants aged 6 months and below. More than half of the children were concomitantly diagnosed clinically for malaria 55 (55.6%). Few had underlying conditions like previous wheezing 9 (9.1%), chronic lung disease 8 (8.1%), low birth weight 9 (10.3%), and 19 (19.4%) were born premature (Table 1). 71/84 (84.5%) children were on mixed feeding (breast milk and bottle-feeding).

The most frequent clinical signs/symptoms were fever, cough, wheezing, difficulty breathing, vomiting, and inability to drink or breastfeed. The clinical presentation of the RSV-positive children was not significantly different from children with RSV-negative acute respiratory infections. 91.3% of RSV-associated ARI patients were wrongly prescribed antibiotics. All of the children with RSV infection survived.

Table 1 shows RSV IgM seropositivity rates by sociodemographic, medical and environmental factors potentially associated with increased risk of RSV IgM seropositivity.

**Fig. 2:**
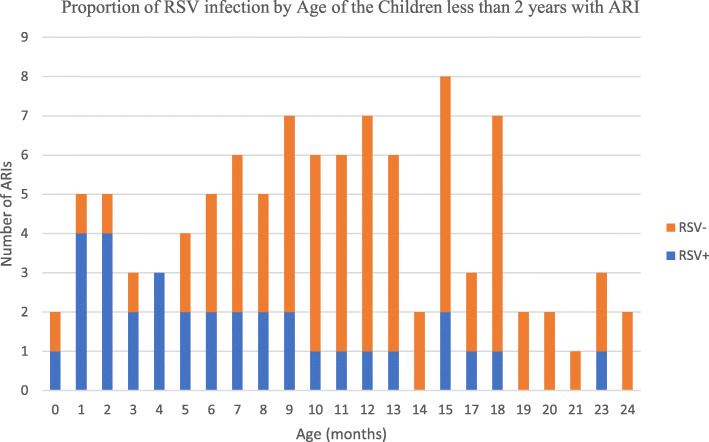
The proportion of RSV infection by the age of the children less than two years with ARI

Of the 18/27 infants aged ≤6 months and presenting with symptoms of ARI were RSV-IgM positive in contrast to the older children up to 2 years were the positivity rate was 15/73 (Fig. 2). The likelihood that the cause for ARI in children ≤6 months is an RSV infection is significantly higher than that for older children [OR = 7.54 (95%CI: 2.62–23.36); *p* = 0.000] than those above 6 months. The RSV IgM prevalence decreased with increasing age.

Twenty-four percent (13/55) of the children with ARI who were clinically diagnosed to be concomitantly infected with malaria but 20/44 who had not tested positive for RSV infection tested RSV IgM positive [OR = 0.38 (0.14, 0.95), *p* = 0.03)]. Furthermore, Table 1 shows that 9/13 children who were exclusively breast fed but only 20/71 children under mixed feeding tested RSV IgM positive [OR = 5.6 (95%CI: 1.37–27.82)] than those on exclusive breastfeeding (Table 1). These relationships were both significant. None of the other sociodemographic, medical or environmental factors was associated with an increased likelihood of RSV IgM positivity in children with ARI.

## Discussion

This is the first multicenter study to better understand the burden of RSV-associated ARI in Cameroon in children below the age of 2 years. The prevalence of 33% recorded is similar to studies from Turkey, Iran, Brazil, and Egypt [11]. Our result differs substantially from that of a previous study conducted in 2009 in the central region of Cameroon showing a rate of detection of RSV of 5.7% in patients with influenza-like illness visiting influenza surveillance centers [15]. One possible reason was that we included all ambulatory or hospitalized cases different from the previous study that included only outpatients and included children and adults. Another possible reason could be that this study was conducted in the late phase of the rainy season and maybe a decreasing period of the RSV season in Cameroon [15]. Another study by Kenmoe et al. 2013 showed that RSV was the second most common respiratory virus (13.3%) among hospitalized children ≤15 years of age with severe acute respiratory infections (SARI) following human adenovirus in Yaoundé, Cameroon and unusually before influenza [16].

This study showed that the most likely cause for ARI in children less than 6 months is an RSV infection was significantly higher than for older children. This means a significant RSV disease burden among children below 6 months which is in-line with other researchers [18]. It suggest the necessity of passive protection against RSV infection at birth, either through maternal immunization or administration of a birth dose of an affordable and extended half-life monoclonal antibodies at birth at least in at risk infants. It also highlights again the importance of developing vaccines for active infant immunization to provide durable protection against RSV disease.

Though the male-female ratio of RSV-positive patients was 1:2, gender was not a significantly associated factor in our study. This result is similar to a prevalence study in Brazil [19]. Nevertheless, some studies in the literature have revealed male sex as a risk factor to acquire RSV infection, but this observation is currently not well defined [18–20].

Twenty-four percent of the children with ARI who were clinically diagnosed to be concomitantly infected with malaria were tested RSV IgM positive. This finding is similar to that of Sricharoenchai et al. 2016 which showed malaria co-occurrence to RSV associated respiratory tract infection [11]. This is an important finding and we encourage further exploration. Children were only clinically diagnosed with malaria in this study. Malaria infection is suspected in all patients with fever. And may result in fever and raised respiratory rate; therefore, it could be an infection mimicking ALRI in countries where malaria is endemic. Increasing awareness is essential for healthcare workers who should provide adequate diagnosis and treatment of both acute respiratory infections and malaria.

Ninety-one percent of the RSV-positive children were wrongly prescribed antimicrobial drugs (antibiotics). Antibiotics were not indicated for treatment as these were viral RSV infection. This finding was similar to that of a study in Saudi Arabia that demonstrated a high prevalence of antibiotic misuse ranging from 42 to 92%, especially in children [21]. Several factors associated with antibiotics overuse include cultural, behavioral, socioeconomic and educational level [21]. The overuse of antibiotics has been associated to accelerate antimicrobial resistance (AMR) which remains a major public health challenge. Over prescription of antibiotics by clinicians is a primary care issue, where most infections are of viral origin, especially respiratory tract infections. Multifaceted strategies to mitigate the overuse of antibiotics are successful and stronger than one program [22]. A global strategic initiative is needed to build a portfolio of vaccines targeting AMR [21].

According to the literature, infants with underlying medical conditions (prematurity and low birth weight), poor parental education, exposure to household smoking and to indoor air pollution have been revealed as risk factors to acquire RSV infections [18, 23]. Still, none of the elements were shown as significant associated factors in our study. Further large cohort and interventional studies in LMIC settings are needed to elucidate these risk factors, as these studies are best suited for this purpose. Parents and guardians who do not have appropriate knowledge of prevention steps place children at higher risk. The home atmosphere of the child is also important. Children from low-income families tend to be more at risk, possibly in part due to lack of access to primary services and lack of maternal education. Exposure to passive tobacco smoke is one of the most important environmental risk factor for infant respiratory infection [18]. A case-control analysis of 53 children with bronchiolitis found that the best indicator was some exposure to passive smoke (*p* = 0.004) [18]. The study sites of our research are located in the rural and semi-rural settings in the coastal regions of Cameroon, where most of the families use solid fuel like firewood or charcoal for cooking. Exposure to household air pollution can be avoided and this is possible even in low-income settings if provided with appropriate resources.

Our finding showed that children on exclusive breastfeeding were more likely to acquire an RSV infection than those on mixed feeding. However, the literature revealed that breastfeeding can reduce the frequency, severity, and mortality of respiratory disease in infants [24]. We encourage further exploration in this LMIC context.

Among the RSV-positive children, the most frequent clinical symptoms/signs were fever, cough, wheezing, difficulty breathing, vomiting, and inability to drink or breastfeed. This is the typical symptomatology of acute respiratory infection; RSV is one of the etiologies indicating, therefore, that the healthcare system in Cameroon and other LMICs should put in place diagnostic possibilities in their health facilities to guide management and infection control. This also shows that emphasis should be placed on prevention. An RSV vaccine will play a vital role in reducing the infant mortality that is highest in low- and middle-income countries.

A third of the RSV-positive children were clinically presented with severe bronchiolitis, and one-sixth with pneumonia as diagnosed by the attending physicians. This indicates that RSV is an established cause for acute lower respiratory infections in children. In settings where viral etiologies are not systematically checked and resources not available, the critical solution to save lives is through preventive measures like vaccines and monoclonal antibodies.

The strength of our study lies in the uniqueness of conducting such a seroepidemiological study in these rural and semi-rural communities in the Littoral region of Cameroon faced with several human resources, infrastructural and logistical bottlenecks. The study sites were mostly first-line primary and secondary healthcare facilities with little experience in health care research. This study was integrated into their routine health care activities and has undoubtedly built a clinical research capacity that needs to be further strengthened in such settings.

A limitation of our study was that we relied on a single laboratory test for detecting RSV antigen, the IgM RSV ELISA test. This might have led to underestimates or overestimates of the disease burden. Compared to virus isolation, using the ELISA test to detect RSV has been shown to have a sensitivity of 94% and a specificity of 97%. There is also the possibility of higher false positives as compared to reverse transcription polymerase chain reaction (RT-PCR) which is the standard test. Still, we limited this by using IgM RSV ELISA, which has a life span of just 30 days in the immune system and also included only children with acute respiratory infections with an early onset of not more than 7 days.

## Conclusions

RSV burden is high among children less than 2 years with ARI in the Littoral region of Cameroon. Accurate clinical and laboratory diagnosis of RSV infection among these patients with ARI would be necessary to reduce the disease burden, large-scale RSV spread, and the misuse of antimicrobial drugs. Further studies are required to better understand antimicrobial drug overuse and abuse, especially in this era of antimicrobial resistance. There is a need for an effective public health RSV surveillance system with standard laboratory techniques and equipment to better understand the RSV disease age-specific incidence, seasonality, and RSV burden among patients in the communities in Cameroon.

## Data Availability

The datasets used and/or analysed during the current study are available from the corresponding author on reasonable request.

## Abbreviations

ARI: Acute respiratory infections
ELISA: Enzyme-linked immunosorbent assay
HRP: Horseradish peroxidase
IgM: Immunoglobulin M
LMIC: Low-and middle-income countries
RSV: Respiratory syncytial virus
TMB: Tetramethylbenzidine
